# Editorial: Immune Monitoring Responses in Renal Autoimmune Diseases

**DOI:** 10.3389/fimmu.2021.722791

**Published:** 2021-06-29

**Authors:** Y. K. Onno Teng, Charles Dickson Pusey, Augusto Vaglio, Chi Chui Mok, Cees van Kooten

**Affiliations:** ^1^ Center of Expertise for Lupus-, Vasculitis- and Complement-Mediated Systemic Autoimmune Diseases, Department of Internal Medicine—Nephrology Section, Leiden University Medical Center, Leiden, Netherlands; ^2^ Centre for Inflammatory Disease, Department of Immunology and Inflammation, Imperial College London, Hammersmith Hospital, London, United Kingdom; ^3^ Department of Biomedical, Experimental and Clinical Sciences “Mario Serio”, University of Firenze, and Nephrology and Dialysis Unit, Meyer Children’s Hospital, Firenze, Italy; ^4^ Division of Rheumatology, Department of Medicine, Tuen Mun Hospital, Hong Kong, Hong Kong

**Keywords:** Gl****omerulonefritis, renal autoimmune diseases, Lupus Nephritis, ANCA - associated vasculitis, membranoproliferative glomerulonephritis (MPGN)

Exciting times for the field of renal autoimmune diseases have begun. In 2021, for the first time, two new drugs belimumab (1) and voclosporin (2) are approved for the treatment of lupus nephritis (LN) (1, 2). Other novel targeted therapies demonstrate clinical efficacy in large, randomized trials, such as avacopan for ANCA-associated vasculitis (AAV) (3), imlifidase for Goodpasture’s disease and iptacopan for IgA nephropathy (IgAN). Pathogenic molecules are specifically targeted by new drugs that help to uncover novel aspects of disease mechanisms leading to glomerulonephritis. Simultaneously, the field is boosted by novel big data technologies on the single cell levels such as high-sensitive multi-color flow cytometry, single-cell genomics (single-cell RNA sequencing - scRNAseq), single cell metabolomics and proteomics. The novel treatment options in renal autoimmune diseases almost simultaneously require new immunomonitoring tools. ‘Immunomonitoring’ includes the wide range of approaches to monitoring immune responses by the cellular immune system (e.g. T-cells, B-cells, plasma cells, dendritic cells, neutrophils etc.), or by the humoral immune system (e.g. cytokines, (auto-)antibodies, urinary markers, etc.). Monitoring relevant immune responses in patients with renal autoimmune diseases helps us to better understand a) the underpinning immunological pathophysiology of these diseases; b) the beneficial effects of novel treatments on autoimmunity; and c) can potentially help doctors and patients guide a personalized treatment strategy, adding information on immunological (non-)response to a clinical (non-) response treatment and on disease prognosis. In the present Research Topic, we have been able to collect for you a vast amount of research addressing novel ways and the role of immunomonitoring in the broad range of renal autoimmune disease including LN, AAV, IgAN, idiopathic membranous nephropathy (iMN) and complement-mediated disease (CMD).

## Immunomonitoring in Lupus Nephritis

A review by professor Rekvig sets the stage for this Research Topic by eluding to the fundaments of immunomonitoring in SLE/LN. It addresses the issue whether, from a principal point of view, anti-dsDNA antibodies can be accepted as a clinical biomarker for SLE, without clarifying what we define as an anti-dsDNA antibody, and in which biologic contexts these antibodies appear.

The group of Wu et al. addressed the epitope spreading of anti-C1q autoantibodies in LN with regard to the “anti-C1q A08 antibodies”. These are part of the anti-C1q antibody family, which recognize nearly complete cryptic epitope in ELISA. Of note, these anti-C1q antibodies recognize the exposed epitope of C1q coated on an ELISA plate. C1q A08 was demonstrated to be important for activation of classical complement pathway and is an important contributor to pathologically relevant anti-C1q antibodies in LN.

The group of Hong et al. provided an overview of the recent advances in our understanding of renal tubular epithelial cells in LN, and the potential role of tubular epithelial cells as biomarkers in the diagnosis, prognosis, and treatment of LN, as well as the future therapeutic potential of targeting the tubulointerstitium for the treatment of patients with LN. In line with this review, several newly identified urine biomarkers, including monocyte chemoattractant protein-1 (MCP-1), neutrophil gelatinase associated lipocalin (NGAL), TNF-like WEAK inducer of apoptosis (TWEAK), and vascular cell adhesion molecule-1 (VCAM), are proteins that may arise directly from inflamed kidneys and have been promising in monitoring LN disease status. In this Research Topic, the group of Zhang et al. demonstrated that the urinary biomarker sCD163 outperformed C3, C4, urinary protein-to-creatinine ratio, or anti-dsDNA antibody in discriminating non-proliferative LN class II or V from proliferative LN class III or IV. A promising finding that can help identifying active LN patients in whom a renal biopsy could be avoided.

Lastly, two studies in this Research Topic address cell-directed immunomonitoring in LN. With respect to T-cells, the group of Ye et al. demonstrated significant differences in TCR diversity and usage of TRBV/TRBJ genes between SLE patients and healthy controls, identifying a set of signature V–J combinations characteristic for their SLE cohort. Further research in this field may facilitate the development of novel immune biomarkers. With respect to B-cells, the group of Yap et al. explored B-cell subsets demonstrating an exhaustion of the naïve B-cell subset in the most active and relapsing LN patients. This observation relates to the phenomenon of ‘B-cell hyperactivation’ in LN and the group demonstrated that microRNA-148a is an important mediator and might be a potential therapeutic target in LN.

## Immunomonitoring in iMN

The group of Liu et al. reviews the striking low quantity of inflammatory cells in the kidney of iMN patients, related to the immune responses leading to anti-podocyte IgG4 autoantibodies in iMN. The expanding spectrum of anti-podocyte antibodies identified in iMN patients is reviewed by the group of Tesar and Hruskova.

Both reviews set the stage for the findings by the group of Cremoni et al. who demonstrated a dysregulated cytokine balance skewed towards Th17 immune responses in iMN, with worse prognosis with more relapses and thromboembolic events.

Lastly, the group of Huang et al. demonstrated anti-PLA2R autoantibodies in patients with autoimmune thyroid disease, notably Hashimoto hypothyroiditis, which is associated with proteinuric AutoImmune Thyroid Disease (AITD) associated nephropathy in 10% of the cases. Given the commonality of anti-PLA2R autoantibodies in these overlapping disease, this common pathologic substrate may help identifying those patients that could profit from immunosuppressive or B-cell targeted therapies.

## Immunomonitoring in ANCA-Associated Vasculitis

Monitoring the immune responses in AAV patients is very much directed at dissecting the underpinning mechanisms of anti-neutrophil cytoplasmic autoantibodies (ANCAs). The group of Granel et al. reflects on the Kurlander effect of PR3-ANCA autoantibodies and the arguments supporting the existence of pathogenic as well as non-pathogenic PR3-ANCAs, which is a relevant concept when employing immunomonitoring in AAV. Subsequently, the group of Thompson et al. investigated serial measurements of PR3-ANCA in AAV patients and their relation to non-response to induction treatment (or vice versa, achieving remission to treatment) and prediction of future relapses In conjunction, the group of van Dam et al. demonstrated a strong reduction in circulating B-cells after treatment of AAV patients with the B-cell depleting agent rituximab (RTX). However, when employing high-sensitivity flow cytometry as an immunomonitoring tool, RTX treatment never resulted in complete B-cell depletion and residual ANCA-specific memory B-cells remained detectable which opens a whole, new field of immunomonitoring in AAV patients undoubtedly warranting further research.

## Immunomonitoring In Complement-Mediated Diseases, Iga Nephropathy And Other Renal Autoimmune Diseases

With respect to IgAN, the group of Selvaskandan et al. provides a truly complete overview of the immense progress made in identifying and validating new biomarkers to facilitate personalization of prognostication and treatment of IgAN.

With respect to CMDs, the group of Gao et al. reviewed the dual roles of the C3a/C3aR interactions, that can exert anti-inflammatory or pro-inflammatory effects depending on the type of kidney disease, with the aim of understanding in-depth its controversial roles and its potential therapeutic value.

Lastly, the group of Ramponi et al. reviewed the current literature to provide guidance on serum biomarkers that could support further testing for kidney involvement in primary Sjögren syndrome (pSS). Patients with pSS typically present with proximal renal tubular acidosis, distal renal tubular acidosis and/or acute (on chronic) kidney impairment for which the most classical kidney lesions are tubulointerstitial nephritis (TIN) and membranoproliferative glomerulonephritis (MPGN).

## Conclusion

The present Research Topic aims to foster knowledge and discussion on investigating and understanding relevant pathological mechanisms underpinning renal autoimmune diseases. As such, these collective research efforts strengthen our Immuno-nephrology community’s capabilities to translating the wealth of data, mostly derived from recent technological advances, into useful discoveries for clinical applicability. Ultimately, the monitoring of the relevant immune phenomena in patients with renal autoimmune diseases will lead to useful intervention to influence disease progression with effective targeted drugs.

## Author Contributions

YT and CK prepared the manuscript. All authors contributed to the article and approved the submitted version.

## Funding

The work of YT is supported by the Dutch Kidney Foundation (17OKG04) and the Netherlands Scientific Organisation.

## Conflict of Interest

The authors declare that the research was conducted in the absence of any commercial or financial relationships that could be construed as a potential conflict of interest.
